# Metabolic and Post-Translational Vulnerabilities of Glioblastoma: Disulfidptosis, Glycosylation, and Implications for CAR-T Therapy

**DOI:** 10.3390/cells15121087

**Published:** 2026-06-15

**Authors:** Tadeusz Strózik, Adrianna Rutkowska, Tomasz Wasiak, Damian Ciunowicz, Piotr Rieske, Natalia Szczepaniak, Ewelina Stoczyńska-Fidelus

**Affiliations:** 1 Department of Molecular Biology, Chair of Medical Biology, Medical University of Lodz, Zeligowskiego 7/9 St., 90-752 Lodz, Poland; adrianna.rutkowska@umed.lodz.pl (A.R.); tomasz.wasiak@umed.lodz.pl (T.W.); damian.ciunowicz@umed.lodz.pl (D.C.); natalia.szczepaniak@student.umed.lodz.pl (N.S.); ewelina.stoczynska-fidelus@umed.lodz.pl (E.S.-F.); 2 Department of Research and Development, LEK-AM Pharmaceutical Company Ltd., Inwestycyjna 7 St., 95-050 Konstantynow Lodzki, Poland; piotr.rieske@umed.lodz.pl; 3 Department of Tumor Biology, Chair of Medical Biology, Medical University of Lodz, Zeligowskiego 7/9 St., 90-752 Lodz, Poland; 4 Student Scientific Circle at the Department of Molecular Biology, Chair of Medical Biology, Medical University of Lodz, Zeligowskiego 7/9 St., 90-752 Lodz, Poland

**Keywords:** glioblastoma, disulfidptosis, SLC7A11/xCT, redox homeostasis, metabolic plasticity, glycosylation, glycocalyx, antigen accessibility, antigen shedding, CAR-T cell therapy

## Abstract

**Highlights:**

**What are the main findings?**

- The review identifies disulfidptosis as an experimentally supported, context-dependent form of redox-dependent cell death in glioblastoma and places it within the broader metabolic–redox vulnerabilities of this disease.
- It summarizes how glycosylation-driven epitope masking, antigen shedding, and glycocalyx remodeling may impair CAR-T-cell recognition, highlighting functional epitope accessibility, in addition to antigen expression, as a key determinant of therapeutic efficacy.

**What are the implications of the main findings?**

- Modulating tumor glycosylation, glycocalyx architecture, or antigen shedding may enhance CAR-T efficacy by improving epitope accessibility and preserving surface target availability.
- Engineering CAR constructs to recognize glycan-accessible or less glycan-shielded epitopes and leveraging glycosylation profiles as biomarkers may improve patient stratification and therapeutic outcomes. In contrast, the therapeutic relevance of combining disulfidptosis-oriented interventions with CAR-T cells in glioblastoma remains to be established.

**Abstract:**

Glioblastoma (GB) remains one of the most therapy-resistant solid tumors, characterized by profound metabolic plasticity, intratumoral heterogeneity, and a highly immunosuppressive microenvironment. While immunotherapies such as chimeric antigen receptor T (CAR-T) cells have shown promise in hematological malignancies, their efficacy in GB has been limited. Emerging evidence suggests that tumor-specific metabolic dependencies and post-translational modifications (PTMs) may represent exploitable vulnerabilities. This review discusses disulfidptosis, a recently described form of regulated cell death driven by disulfide stress under conditions of limited reducing capacity, as a context-dependent metabolic–redox vulnerability in GB. We further discuss how altered protein glycosylation and glycocalyx architecture in glioblastoma regulate cell survival, death signaling, and immune recognition. Particular emphasis is placed on the glycosylation of surface antigens targeted by CAR-T cells, including EGFR/EGFRvIII, IL-13Rα2, mesothelin, B7-H3, HER2, and GD2, and on how glycan-dependent epitope accessibility may limit therapeutic efficacy. Finally, we distinguish disulfidptosis, whose direct relevance to CAR-T-cell responses remains to be established, from glycosylation and glycocalyx remodeling as more direct determinants of target–antigen accessibility and immune recognition. Therapeutic strategies addressing these vulnerabilities may provide rational opportunities to improve CAR-T-based and combinatorial therapies for GB.

## 1. Introduction

Glioblastoma (GB) remains the most aggressive primary malignant brain tumor in adults. Despite standard treatment consisting of maximal safe resection followed by radiotherapy and temozolomide, recurrence is almost inevitable, underscoring that therapeutic resistance is deeply embedded in the biology of the disease rather than arising solely from technical limitations of treatment [1]. A central determinant of this resistance is profound intratumoral heterogeneity. Single-cell and integrative transcriptomic studies have shown that malignant cells within the same GB differ markedly in proliferative capacity, hypoxia-associated programs, oncogenic signaling, and stemness-related features, while also retaining substantial plasticity in response to genetic drivers and microenvironmental constraints [2,3].

Within this context, metabolic reprogramming in GB should not be viewed merely as a consequence of rapid proliferation, but as an active component of tumor adaptation. Metabolomic and functional studies have demonstrated broad rewiring of central carbon metabolism, increased anabolic activity, and flexible substrate utilization in GB, reflecting a high degree of metabolic plasticity [4,5,6,7].

This adaptability supports not only biomass production but also survival under fluctuating nutrient availability, oxidative stress, and therapeutic pressure. In particular, altered glucose metabolism in GB extends beyond enhanced glycolysis and contributes to biosynthetic fitness and treatment resistance, whereas glutamine metabolism can serve as a compensatory pathway that sustains growth and nucleotide synthesis under stress conditions [5,8,9,10,11,12].

A particularly relevant metabolic node in this setting is the cystine/cysteine–redox axis. Overexpression of the system $x_{c}^{-}$ transporter, including its light-chain subunit *SLC7A11* (xCT), has been associated with an altered metabolic state, increased resistance to oxidative stress, and reduced sensitivity to temozolomide in glioblastoma [13]. More broadly, elevated *SLC7A11* activity has been shown to increase cancer-cell dependence on glucose, thereby creating a mechanistic basis for context-dependent vulnerability under glucose-limiting conditions [14]. In GB models, cystine uptake through xCT during glucose deprivation promotes rapid NADPH depletion, reactive oxygen species accumulation, and cell death, whereas adaptive downregulation of xCT can improve survival under the same conditions [15,16]. This conditional vulnerability provides the metabolic context in which disulfidptosis can be induced in GB under defined conditions.

Within this review, these vulnerabilities are considered as related but mechanistically distinct components of GB adaptation. Disulfidptosis represents a context-dependent metabolic–redox vulnerability that can be induced in GB under defined conditions, whereas altered glycosylation and glycocalyx remodeling may more directly influence antigen accessibility and immune evasion. In this framework, failure of CAR-T-cell therapy may depend not only on which antigens are expressed, but also on whether they remain functionally accessible at the tumor-cell surface.

## 2. Metabolic and Redox Context of Disulfidptosis in Glioblastoma

Glioblastoma develops within a microenvironment characterized by fluctuating oxygen and nutrient availability and therefore relies on adaptive metabolic and redox-buffering programs. In experimental GB models, cycling hypoxia promotes radioresistance through NADPH oxidase 4 (NOX4)-dependent reactive oxygen species (ROS) signaling, whereas ROS-mediated activation of the HIF-1α/NF-κB/Bcl-xL axis reduces responsiveness to temozolomide [17,18]. These findings indicate that redox adaptation is an important component of treatment resistance in GB.

Among the metabolic processes contributing to redox homeostasis, glucose metabolism is particularly relevant to disulfidptosis. Hexokinase 2 (HK2) supports aerobic glycolysis and tumor growth in human GB [8], while recent in vivo evidence indicates that gliomas redirect glucose-derived carbon toward biosynthetic pathways supporting tumor expansion [9]. The oxidative branch of the pentose phosphate pathway (PPP) is central in this context because it maintains NADPH-dependent redox buffering. In glioma models, Nrf2-dependent regulation of *TERT* and HSPB1/SIRT2-mediated activation of glucose-6-phosphate dehydrogenase (G6PD) have been linked to PPP activity, NADPH availability, and resistance to oxidative or DNA-damaging stress [19,20]. Accordingly, the glucose–PPP–NADPH axis provides the most direct metabolic background for understanding redox-dependent susceptibility to disulfidptosis.

A central node within this axis is the cystine/glutamate antiporter system $x_{c}^{-}$ and its light-chain subunit SLC7A11/xCT. In GB, increased system $x_{c}^{-}$ expression alters the metabolic state of tumor cells, enhances resistance to oxidative stress, and reduces sensitivity to temozolomide [13]. More broadly, increased *SLC7A11* activity may create a dependency on glucose-driven reducing capacity because continuous cystine uptake requires NADPH-dependent reduction of cystine to cysteine [14]. Consistently, in GB cells, xCT-mediated cystine uptake under glucose deprivation promotes NADPH depletion and cell death, whereas high-cell-density-associated downregulation of xCT improves survival under the same metabolic conditions [15,16]. Thus, cystine uptake can shift from a redox-protective adaptation to a metabolic liability when glucose-dependent reducing capacity becomes insufficient.

**Figure 1:**
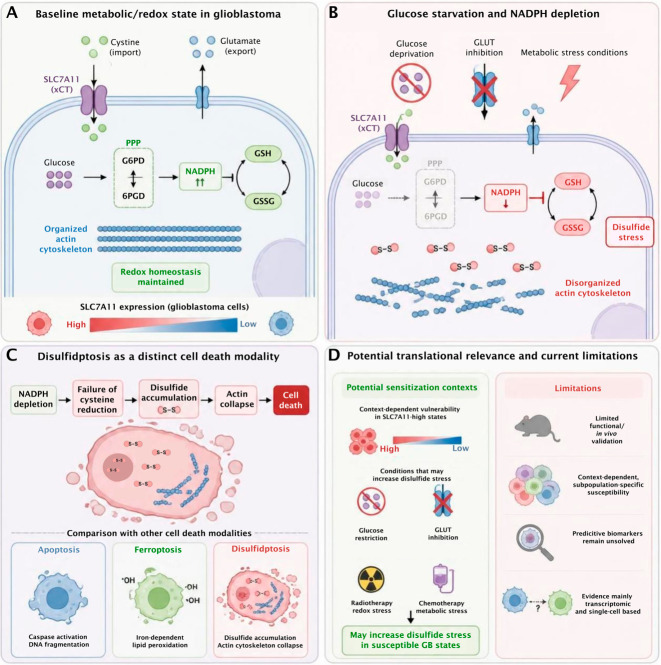
Conceptual framework of metabolic and redox constraints potentially shaping disulfidptosis susceptibility in glioblastoma. Panel (**A**) shows the adaptive metabolic/redox state of GB cells, in which glucose flux through the pentose phosphate pathway (PPP) supports NADPH production, glutathione balance, and actin cytoskeleton integrity, with heterogeneous SLC7A11/xCT expression across tumor cell subpopulations. Panel (**B**) illustrates metabolic stress, where glucose deprivation, GLUT inhibition, or related constraints reduce PPP-dependent NADPH availability despite continued cystine uptake, promoting disulfide accumulation and cytoskeletal disorganization in SLC7A11/xCT-expressing cells. Panel (**C**) presents disulfidptosis as a distinct regulated cell death mechanism characterized by disulfide stress and actin cytoskeleton collapse, alongside simplified comparisons with apoptosis and ferroptosis. Panel (**D**) summarizes the potential translational relevance and current limitations of disulfidptosis in GB, including context-dependent vulnerability in SLC7A11-high states and possible sensitization by metabolic or therapy-associated redox stress. Abbreviations: 6PGD, 6-phosphogluconate dehydrogenase; GB, glioblastoma; G6PD, glucose-6-phosphate dehydrogenase; GLUT, glucose transporter; GSH/GSSG, reduced glutathione/oxidized glutathione; NADPH, reduced nicotinamide adenine dinucleotide phosphate; PPP, pentose phosphate pathway; SLC7A11/xCT, solute carrier family 7 member 11 (xCT), the light-chain subunit of the system $x_{c}^{-}$ cystine/glutamate antiporter.

Importantly, this vulnerability has now been experimentally linked to disulfidptosis in GB. Tang et al. demonstrated that thioredoxin reductase 1 (TrxR1) inhibition under glucose-starved conditions induces disulfidptosis in GB cells, characterized by disulfide-dependent disruption of the actin cytoskeleton. In an orthotopic GB xenograft model, TrxR1 depletion reduced tumor growth and prolonged survival [21]. These findings establish disulfidptosis as an experimentally demonstrated, context-dependent metabolic–redox vulnerability in GB. Figure 1 summarizes the relevant glucose–PPP/NADPH–cystine/xCT–disulfide stress axis and provides the conceptual framework for the subsequent comparison of disulfidptosis with other forms of regulated cell death.

### 2.1. Disulfidptosis as a Distinct Form of Regulated Cell Death

Disulfidptosis is a recently defined form of regulated cell death driven by excessive intracellular disulfide stress. It was originally defined in SLC7A11-high cancer cells exposed to glucose starvation, in which continued cystine uptake occurs despite insufficient NADPH-dependent reducing capacity. Under these conditions, cystine reduction becomes impaired, intracellular disulfides accumulate, and aberrant disulfide bonding develops in actin cytoskeletal proteins, ultimately leading to collapse of the F-actin network [22,23].

This mechanism distinguishes disulfidptosis from other major forms of regulated cell death. In apoptosis, cell death is executed primarily through caspase activation downstream of mitochondrial or death receptor signaling. Ferroptosis is characterized by iron-dependent lipid peroxidation and failure of antioxidant systems such as the glutathione/GPX4 axis. Necroptosis depends on RIPK1/RIPK3/MLKL-mediated membrane disruption, whereas pyroptosis is driven by gasdermin-mediated membrane pore formation and inflammatory cell lysis. In contrast, the defining lesion of disulfidptosis is disulfide-stress-induced collapse of the actin cytoskeleton rather than caspase activation, lipid peroxidation, kinase-driven membrane rupture, or gasdermin pore formation [22,23,24].

The relationship between disulfidptosis and ferroptosis is particularly relevant because SLC7A11 may exert opposite effects depending on metabolic context. Under nutrient-replete conditions, increased SLC7A11 activity supports cystine uptake and glutathione synthesis, thereby limiting ferroptotic susceptibility. Under glucose-limited conditions, however, continued cystine uptake increases demand for reducing equivalents and can promote disulfide stress and disulfidptosis when NADPH-dependent reducing capacity becomes insufficient [22,23,25].

The actin cytoskeleton is not merely a downstream consequence of redox disturbance but a central determinant of disulfidptosis. The original mechanistic study showed that loss of components of the WAVE regulatory complex suppresses disulfidptosis, whereas Rac activation enhances susceptibility, indicating that active cytoskeletal remodeling contributes to execution of this death program. Furthermore, the phenotype is suppressed by reducing agents and enhanced by thiol-oxidizing compounds, supporting disulfide stress rather than nonspecific oxidative injury as its principal mechanistic driver [22,23].

Disulfidptosis should therefore be considered a distinct redox-dependent form of regulated cell death centered on cytoskeletal disulfide damage. Its defining features and major differences from other regulated cell death modalities relevant to glioblastoma are summarized in Table 1.

### 2.2. Disulfidptosis in Glioblastoma: Experimental Evidence, Heterogeneity, and Translational Limitations

The relevance of disulfidptosis to GB is supported by both experimental and transcriptomic evidence, although these two levels of evidence should be clearly distinguished. Mechanistically, Tang et al. demonstrated that inhibition of TrxR1 under glucose-starved conditions induces disulfidptosis in GB cells, with disulfide-dependent disruption of the actin cytoskeleton. In an orthotopic GB xenograft model, TrxR1 depletion reduced tumor growth and prolonged survival [21]. These findings provide direct experimental evidence that disulfidptosis can be induced in GB under defined metabolic and redox constraints.

Complementary transcriptomic studies indicate that disulfidptosis-related states are heterogeneous across glioma and GB contexts. Fan and Chen used single-cell RNA sequencing and spatial transcriptomics to identify variation in disulfidptosis-related scores among glioma-associated cell populations, with higher scores associated with recurrent disease and less favorable survival outcomes [29]. In parallel, prognostic studies based on disulfidptosis-related gene signatures have linked these transcriptional programs to survival, molecular features of aggressive glioma, and immune infiltration [30,31,32]. These studies support the clinical and biological relevance of disulfidptosis-related expression patterns, but they should not be interpreted as evidence that all high-score tumor subpopulations undergo canonical disulfidptosis in vivo.

A potential link with the immune microenvironment has also emerged. Shu and Li reported that a disulfidptosis–T-cell-exhaustion-associated signature was enriched in endothelial cells and related to angiogenesis, extracellular matrix organization, and immune cell interactions in GB [33]. Their cell-based validation using LN229 cells showed that *SMC4* silencing altered migratory behavior, chemotherapy sensitivity, oxidative stress-associated parameters, and CD4/CD8 T-cell activation. Similarly, Zhou et al. identified a disulfidptosis-related immune checkpoint signature associated with poor outcome and an immunosuppressive GB microenvironment characterized by angiogenesis-, extracellular-matrix-, and T-cell-exhaustion-associated features [34]. These findings suggest that disulfidptosis-related programs may be embedded within broader immunosuppressive tumor states; however, they remain predominantly associative and do not demonstrate improved antitumor immunity following induction of disulfidptosis.

From a translational perspective, disulfidptosis should therefore be considered a context-dependent vulnerability rather than a universal feature of GB. Candidate determinants of susceptibility include SLC7A11 expression, cystine dependence, glucose availability, TrxR1 activity, and the broader redox–metabolic state of tumor cells [21,22,23,35]. Further studies are required to define predictive biomarkers, determine therapeutic selectivity, and evaluate whether disulfidptosis-inducing interventions can be safely combined with existing treatment modalities.

Importantly, although disulfidptosis-related programs have been associated with immune dysfunction and T-cell-exhaustion-related features in GB, no direct evidence currently demonstrates that inducing disulfidptosis improves CAR-T-cell-mediated tumor control in this disease [33,34,35,36]. Accordingly, in the context of CAR-T resistance, disulfidptosis should be viewed as an emerging and hypothesis-generating metabolic vulnerability, whereas the subsequent sections focus on glycosylation and glycocalyx remodeling as more direct determinants of target-antigen accessibility, immune recognition, and CAR-T-cell efficacy.

Metabolic adaptation in GB not only regulates susceptibility to redox-dependent cell death but also influences post-translational remodeling of cell-surface proteins. Among these modifications, glycosylation is particularly relevant to CAR-T-cell therapy because it can alter receptor stability, glycocalyx architecture, and the functional accessibility of tumor-associated antigens.

## 3. Glycosylation as a Metabolic and Post-Translational Regulator in Glioblastoma

Glycosylation plays a central yet underexplored role in GB biology. Unlike protein synthesis, glycosylation is not template-driven but depends on the availability of metabolic substrates and the activity of glycosyltransferases within the ER and Golgi apparatus [37]. As a result, it integrates cellular metabolic state with protein folding, stability, and surface presentation [38].

Metabolic reprogramming in solid tumors shapes critical molecular pathways in GB. This metabolic integration becomes particularly relevant in solid tumors, including GB, where metabolic reprogramming supports rapid proliferation, survival under hypoxia, and therapy resistance [39]. These metabolic adaptations, in turn, affect the hexosamine biosynthetic pathway (HBP), which generates UDP-N-acetylglucosamine (UDP-GlcNAc), a key substrate for N-linked glycosylation, O-linked glycosylation, and O-GlcNAcylation [40]. Through this mechanism, nutrient availability directly influences the structural and functional properties of proteins [41].

Aberrant glycosylation actively shapes tumor progression and heterogeneity in glioblastoma. It modulates receptor signaling, cell adhesion, migration, and immune interactions, and contributes to the formation of a tumor-specific glycocalyx [42]. In GB, these processes are further complicated by pronounced intratumoral heterogeneity and dynamic metabolic adaptation, suggesting that glycosylation patterns are likely context-dependent and variable across cellular subpopulations [43].

### 3.1. Reprogramming of Glycosylation in Solid Tumors

#### 3.1.1. Glycosylation as a Metabolically Driven Process

Glycosylation is tightly linked to cellular metabolism through the HBP, which integrates glucose, glutamine, acetyl-CoA, and nucleotide metabolism to produce UDP-GlcNAc [44]. This metabolite serves as a central donor substrate for multiple glycosylation processes, including N-glycan branching, mucin-type O-glycosylation, and intracellular O-GlcNAcylation [45].

In cancer cells, increased glucose and glutamine uptake enhances flux through the HBP, leading to elevated levels of UDP-GlcNAc [46]. This has direct consequences for glycosylation, as several glycosyltransferases, particularly those involved in higher-order N-glycan branching, are sensitive to substrate availability [47]. As a result, metabolic reprogramming can influence not only the extent but also the structural complexity of glycans attached to proteins [48].

This metabolic dependency positions glycosylation as a functional interface between nutrient availability and protein regulation. Rather than being a static modification, glycosylation reflects the cellular metabolic state and dynamically adapts to environmental conditions, such as hypoxia or nutrient stress [49,50].

#### 3.1.2. N-Linked Glycosylation and Receptor Stability

N-linked glycosylation begins in the ER and continues in the Golgi apparatus, where glycans undergo branching, elongation, and terminal modifications, including sialylation and fucosylation. In solid tumors, these processes are frequently dysregulated, leading to increased complexity and heterogeneity in N-glycan structures [51].

One of the key consequences of altered N-glycosylation is the modulation of membrane protein stability and trafficking. Highly branched N-glycans can enhance receptor retention at the cell surface by reducing endocytosis and promoting interactions with lectins, such as galectins [52]. This prolongs signaling through growth factor receptors, integrins, and other membrane-associated proteins [53].

Importantly, the degree of N-glycan branching is influenced by UDP-GlcNAc availability, linking metabolic flux to receptor stability and signaling intensity. In this context, glycosylation functions as a regulatory layer that fine-tunes oncogenic signaling rather than simply reflecting protein abundance [54].

#### 3.1.3. O-Linked Glycosylation and Glycocalyx Remodeling

O-linked glycosylation, especially mucin-type, creates extended glycans on membrane proteins. In cancer, these glycans become elongated and densely packed, expanding the glycocalyx [55].

Building on these changes, the glycocalyx plays both structural and signaling roles. Notably, its remodeling in solid tumors has been associated with altered cell adhesion, enhanced migration and invasion, and modulation of receptor clustering. Furthermore, a dense glycocalyx can act as a physical barrier, limiting immune cell access to the cell surface and interfering with receptor–ligand interactions [56,57,58,59].

Therefore, changes in O-glycosylation not only affect protein function at the molecular level but also reshape the physical architecture of the cell surface, with direct implications for cell–cell interactions [60].

#### 3.1.4. O-GlcNAcylation as an Intracellular Signaling Regulator

In addition to extracellular glycosylation, UDP-GlcNAc is also used for O-GlcNAcylation, a dynamic intracellular modification of nuclear, cytoplasmic, and mitochondrial proteins. Unlike classical glycosylation, O-GlcNAcylation is reversible and functions analogously to phosphorylation, regulating protein activity, stability, and interactions [61,62].

In cancer cells, increased HBP flux frequently leads to elevated O-GlcNAcylation, which has been linked to enhanced proliferation, stress tolerance, and transcriptional reprogramming [63]. By modulating transcription factors, chromatin regulators, and signaling proteins, O-GlcNAcylation contributes to the maintenance of oncogenic programs [64].

Together, these observations highlight that glycosylation operates on multiple levels, surface and intracellular, and that both are metabolically controlled [65,66].

### 3.2. Glycosylation in Glioblastoma: Current Evidence and Heterogeneity

The characterization of glycosylation in glioblastoma is less advanced than in other solid tumors, yet emerging evidence shows that GB undergoes notable alterations in glycan structures, glycosyltransferase expression, and glycoprotein composition [67,68]. Individual glycosylation regulators play a significant role in glioblastoma biology. For instance, several studies have linked elevated *MGAT1* expression to increased glioma proliferation and migration, partly by stabilizing GLUT1 via N-linked glycosylation. This connects glycosylation to metabolic adaptation and oncogenic metabolism [69,70,71].

Altered sialylation emerges as another key feature of GB biology. Elevated α2,6-sialylation, mediated by ST6GAL1, has been shown to promote tumor growth and sustain brain tumor-initiating cells. Reducing ST6GAL1 impairs sialylation, proliferation, and self-renewal, underlining the role of specific glycan structures in sustaining stem-like phenotypes [67,68].

Hypoxia-driven modulation of glycosylation also influences glioblastoma stem cell maintenance. Specifically, upregulation of GLT8D1 under hypoxic conditions aids glioma stem cell stability by stabilizing CD133 through N-linked glycosylation, demonstrating the impact of the microenvironment on glycosylation and cellular hierarchy [72].

Intracellular O-GlcNAcylation adds a further layer to glycosylation regulation in GB. Increased OGT expression and global O-GlcNAcylation drive tumor cell survival and resistance; OGT inhibition reduces proliferation and induces apoptosis, suggesting a role in therapy resistance [73].

Glycoproteomic analyses reveal profound glycosylation-mediated remodeling of the glioblastoma microenvironment. In particular, changes in the extracellular matrix and cell-surface glycoproteins vary by GB subtype, revealing subtype-specific glycosylation landscapes [37]. Current knowledge about glycosylation heterogeneity in glioblastoma is incomplete. Although molecular heterogeneity is established, high-resolution glycosylation data remain limited, and differences across intratumoral regions and cell types are often inferred rather than directly shown [74].

Collectively, the evidence indicates that glycosylation in glioblastoma is dynamically regulated, metabolically integrated, and crucial to tumor behavior and interactions with the microenvironment. Nevertheless, thorough, spatially resolved glycomic mapping is needed to fully exploit its therapeutic potential [54,74].

These glycosylation changes are not restricted to individual proteins but collectively reshape the cell surface. At the macroscopic level, this is reflected in the remodeling of the glycocalyx, which integrates altered glycoproteins, proteoglycans, and glycolipids into a dense barrier at the tumor-immune interface. Thus, glycosylation-driven glycocalyx remodeling links cellular metabolic state to antigen accessibility and immune evasion [65].

### 3.3. Glycocalyx as a Physical and Signaling Barrier

The glycocalyx of cancer cells is not merely a passive polysaccharide layer, but rather a dynamic and highly organized “glycan scaffold” that redefines the boundary between the cell and its surrounding tumor microenvironment (TME) [57,75,76]. During malignant transformation, including in gliomas, profound remodeling of glycocalyx organization and composition occurs, leading to the emergence of a high-density phenotype (the so-called bulky glycocalyx) [57,75,76,77]. This alteration has both mechanical and signaling consequences, influencing immune recognition, cell survival, and migratory and invasive capacities [57,75,76,77].

A key aspect of this remodeling is the impairment of direct contact between immune effector cells and the tumor cell membrane. The presence of elongated, heavily glycosylated mucins (primarily MUC1 and other MUC-type proteins), along with glycosaminoglycans such as chondroitin sulfate, generates a physical barrier whose thickness exceeds the effective reach of standard T cell receptors (TCRs) [57,75,76,78]. Densely packed, negatively charged sialic acid-containing chains create an environment characterized by elevated osmotic pressure and significant electrostatic interactions, resulting in the repulsion of effector cells and preventing efficient formation of the immunological synapse [57,75,76,78,79]. Consequently, TCR-mediated activation signals are attenuated, allowing tumor cells to evade immune surveillance.

The expanded glycocalyx also impacts mechanotransduction. Mechanical tension generated by this “sugar coat” can induce conformational activation of integrins independently of classical ligand binding to the extracellular matrix (ECM) [57,75,76]. Such ligand-independent activation enhances pro-proliferative and pro-survival signaling pathways, including focal adhesion kinase (FAK) and the PI3K/Akt pathway [57,75,76,77,80]. In glioblastoma, a thickened and tensioned glycocalyx establishes a positive feedback loop with integrin signaling, promoting a mesenchymal, stem-like phenotype associated with therapy resistance and recurrence [75,77,81]. As a result, tumor cells become less responsive to pro-apoptotic and cell cycle-arresting signals present in the TME.

Beyond its mechanical role, the tumor glycocalyx can contribute to immune evasion through inhibitory “don’t eat me” mechanisms that limit phagocytic clearance. In acute myeloid leukemia (AML), Chung et al. identified highly sialylated, mucin-like CD43 as a glyco-immune barrier whose inhibitory function depends on ectodomain length and O-glycan density. Genetic deletion or antibody-mediated blockade of CD43 enhanced macrophage-mediated phagocytosis and increased NK- and T-cell cytotoxicity, demonstrating that glycan architecture itself can suppress both innate and adaptive antitumor immunity [82]. In contrast, in GB, a well-established phagocytic checkpoint is the CD47–SIRPα axis: CD47 expressed on tumor cells engages SIRPα on macrophages and microglia, thereby limiting tumor-cell phagocytosis. In preclinical GB models, anti-CD47 treatment enhanced macrophage-mediated phagocytosis, while disruption of the CD47–SIRPα axis enabled microglia to act as antitumor effector cells [83,84]. Thus, CD43 in AML and CD47 in GB represent distinct but conceptually related mechanisms of escape from phagocytic immune surveillance: the former is directly dependent on a densely sialylated glycan barrier, whereas the latter functions primarily as a receptor-mediated antiphagocytic checkpoint.

The high density of glycans on the cell surface physically shields protein epitopes, including neoantigens, thereby hindering their recognition by both antibodies and TCRs [57,75,76,78,85]. Additionally, hypersialylation—defined as excessive expression and exposure of sialic acid residues—enables tumor cells to mimic glycan patterns typical of healthy tissues [57,76,78,79,86,87]. Sialylated glycans interact with Siglec receptors (sialic acid-binding immunoglobulin-type lectins), present on immune cells such as NK cells and macrophages, delivering inhibitory signals analogous to immune checkpoint pathways [57,76,78,79,85,86,87,88]. Activation of Siglecs promotes the transmission of a “don’t eat me” signal, leading to functional inactivation of effector cells and fostering an immunosuppressive tumor microenvironment. In glioblastoma, altered glycans and their interactions with lectins (including Siglecs, galectins, and C-type lectins) shape an immunosuppressive landscape, promoting myeloid-derived suppressor cells (MDSCs) and M2 macrophages, while suppressing CD8^+^ T cells and dendritic cells [78,85,86,89,90].

Alterations in glycocalyx composition and organization are also closely linked to the migratory and invasive properties of cancer cells. Increased surface hydration, resulting from the abundance of highly hydrated glycosaminoglycans, facilitates cell movement within the dense connective tissue stroma by acting as a form of biological “lubrication” [57,75,76,77]. At the same time, specific glycan structures such as sialyl Lewis X function as ligands for selectins expressed on vascular endothelial cells, supporting rolling, adhesion, and extravasation [85,87,91]. In glioblastoma, such glycocalyx reprogramming promotes characteristic tumor infiltration along blood vessels and nerve fibers, as well as migration through white matter tracts [75,77,78].

At the molecular level, glycocalyx modifications—including alterations in glycosaminoglycan structure, integrin sialylation, and CD44 glycosylation—modulate multiple processes: adhesion to extracellular matrix components (e.g., fibronectin and laminin), migration along the ECM, ECM degradation via regulation of matrix metalloproteinases, and mechanotransduction, i.e., how cells sense and interpret environmental stiffness [57,75,76,77,80]. In highly infiltrative tumors such as glioblastoma, these processes directly translate into enhanced migration along blood vessels and nerve fibers, a hallmark of their aggressive biology [75,77,78].

### 3.4. Glycosylation and Cell Death: Mechanisms of Resistance and the Redox Vulnerability

The relationship between glycosylation and cell death represents one of the most rapidly evolving areas of modern cancer biology [92,93]. These processes bridge classical carbohydrate biochemistry with the regulation of signaling pathways and cellular bioenergetics, influencing both sensitivity to pro-apoptotic stimuli and newly described forms of cell death associated with metabolic and redox stress [40,92,93].

An important aspect is the modulation of death receptors of the TNF family, such as FAS, DR4, and DR5. These glycoproteins require proper N-glycosylation for correct folding, conformational stability, and trafficking to the plasma membrane [92,94,95]. In cancer cells, altered glycosylation profiles of these receptors are frequently observed, leading to their retention in the Golgi apparatus, enhanced proteasomal degradation, or aberrant surface expression [92,94,95,96]. Even when receptors reach the membrane, specific modifications such as core fucosylation may impair or prevent proper oligomerization upon ligand binding (e.g., TRAIL) [94,96,97]. Disruption of functional death receptor complex formation effectively blocks caspase activation and confers resistance to apoptosis induced by both therapeutic agents and immune effector mechanisms [92,94,95,96,97,98]. Furthermore, increased sialylation and branching of N-glycans stabilize receptors in conformations less prone to activation, adding another layer of regulation to the apoptotic response threshold [92,93,95,98]. In GB cells, modulation of DR5—either pharmacologically (e.g., celastrol) or via temozolomide-induced senescence—can significantly enhance sensitivity to TRAIL, highlighting glycosylation and DR5 expression as key determinants of therapeutic response [99,100,101].

Glycosylation also plays a crucial role in regulating the stability and half-life of surface proteins involved in pro-survival signaling, such as EGFR, integrins, and CD44 [40,93,102,103]. Properly and extensively glycosylated proteins are better protected from proteolysis, exhibit slower internalization, and display prolonged retention at the plasma membrane [40,93,102]. Under tumor conditions, this favors the dominance of pro-proliferative and pro-survival signaling over pro-apoptotic cues. Conversely, selective loss of specific glycan structures can direct proteins toward endocytosis and lysosomal degradation, reducing growth-promoting signaling and facilitating the initiation of cell death [40,93,102,103]. Thus, the glycosylation machinery and the endosomal–lysosomal system are functionally coupled in regulating the fate of surface receptors [40,93,102].

Reprogramming of glycosylation in GB may therefore influence both susceptibility to apoptotic signaling and the functional organization of the tumor cell surface. This relationship is particularly relevant to CAR-T-cell therapy because altered glycosylation and glycocalyx remodeling may preserve detectable antigen expression while reducing epitope accessibility, impairing CAR binding and immune synapse formation. Thus, in addition to antigen heterogeneity and antigen loss, biochemical masking and altered surface display of target antigens may contribute to CAR-T-cell resistance in GB [59,75,104,105].

## 4. Why CAR-T Therapy Fails in Glioblastoma

### 4.1. Recognition Principles and Brief Evolution of CAR-T-Cell Therapy

**Figure 2:**
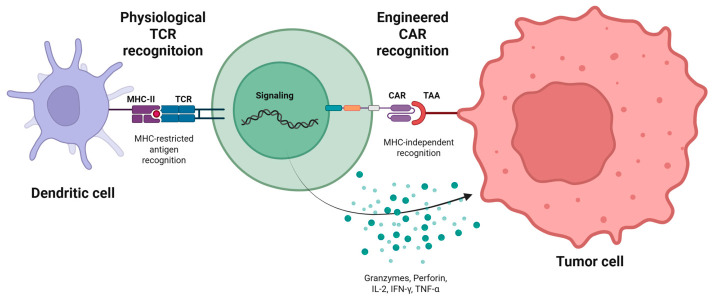
Schematic comparison of conventional TCR-mediated and CAR-mediated tumor-antigen recognition. In conventional T-cell recognition, the T-cell receptor (TCR) engages a peptide–MHC complex displayed by an antigen-presenting or target cell, making recognition dependent on antigen processing and MHC presentation. In contrast, a chimeric antigen receptor (CAR) binds an intact surface tumor-associated antigen (TAA) through an extracellular antibody-derived scFv, independently of peptide processing and MHC presentation. CAR engagement activates intracellular signaling domains and induces cytotoxic mediator release and cytokine secretion toward the tumor cell. Arrows indicate the direction of antigen recognition and cytotoxic effector response, whereas colors are used to distinguish the main cell types and schematic components. Abbreviations: CAR, chimeric antigen receptor; MHC, major histocompatibility complex; scFv, single-chain variable fragment; TAA, tumor-associated antigen; TCR, T-cell receptor.

CAR-T-cell therapy was developed to genetically redirect T lymphocytes toward surface tumor antigens independently of peptide processing and major histocompatibility complex (MHC) presentation (Figure 2). The concept originated from early chimeric receptor designs developed in the late 1980s and early 1990s, initially referred to as “T-body” constructs, in which antibody-derived recognition elements were combined with T-cell signaling components [106,107]. First-generation CARs typically incorporated an antibody-derived single-chain variable fragment (scFv) linked to a single activation module, most commonly CD3ζ, thereby enabling direct recognition of cell-surface antigens without requiring peptide presentation by MHC molecules [107,108]. Although these constructs established the principle of MHC-independent tumor recognition, signaling through a single activation domain was insufficient for optimal T-cell expansion and persistence, and early clinical studies demonstrated limited persistence and modest antitumor efficacy [108,109,110].

The development of second-generation CARs represented a major advance, with the incorporation of an additional co-stimulatory domain, most commonly CD28 or 4-1BB/CD137, together with CD3ζ. These modifications improved T-cell persistence and antitumor activity in preclinical models and subsequently became central features of clinically successful CAR-T-cell products [111,112]. Later-generation strategies have incorporated more complex signaling architectures, cytokine-support systems, safety switches, and programmable or logic-gated recognition modules intended to improve activity and safety, particularly in immunosuppressive solid-tumor microenvironments [113].

The clinical breakthrough of CAR-T-cell therapy was achieved in CD19-positive B-cell malignancies. In 2017, tisagenlecleucel (Kymriah) became the first CAR-T-cell product approved by the U.S. Food and Drug Administration for pediatric and young adult patients with refractory or relapsed B-cell precursor acute lymphoblastic leukemia, followed later that year by the approval of axicabtagene ciloleucel (Yescarta) for adults with relapsed or refractory large B-cell lymphoma [114,115]. However, translating this success to solid tumors, including glioblastoma, remains considerably more challenging. In contrast to TCRs, which recognize peptide–MHC complexes, CARs bind intact surface epitopes; consequently, glycosylation and glycocalyx remodeling may directly influence target accessibility, CAR binding, and immune synapse formation even when target-antigen expression is retained.

### 4.2. Antigen Heterogeneity and Antigen Loss in Glioblastoma

Antigen heterogeneity represents one of the principal barriers to effective CAR-T-cell therapy in GB. It occurs both between patients, who may differ substantially in the expression of candidate target antigens despite the same histopathological diagnosis, and within individual tumors, where only selected malignant subpopulations may express a therapeutically targetable antigen. Consequently, CAR-T cells directed against a single antigen may eliminate antigen-positive cells while leaving antigen-negative or antigen-low populations unaffected.

EGFR and its tumor-associated variant EGFRvIII illustrate this challenge. In the cohort analyzed by Ravanpay et al., EGFR expression was high in 23% of cases, low in 20%, and undetectable in 57% [116]. In a separate analysis of 76 GB tumors with focal EGFR amplification, Francis et al. found frequent co-expression of EGFR alterations: 71% (54/76) expressed EGFRwt together with at least one aberrant EGFR variant, and 30% (23/76) expressed two or more EGFR variants. These non-mutually exclusive observations indicate substantial molecular heterogeneity within EGFR-amplified GB rather than distinct patient categories that should be summed [117]. Single-nucleus sequencing further demonstrated marked intratumoral EGFR heterogeneity, showing that distinct EGFR variants, including EGFRvII, EGFRvIII, and C-terminal deletion variants, may occur in separate tumor subclones within the same lesion [117]. Importantly, EGFRvIII may be present only in a fraction of malignant cells, leaving other tumor-cell populations outside the scope of EGFRvIII-directed therapy. A similar limitation applies to IL-13Rα2, whose expression is heterogeneous across GB tumors and may vary within individual lesions [118].

A further major challenge is antigen loss, defined as a reduction or complete loss of expression of the target antigen recognized by CAR-T cells. This phenomenon may arise through therapeutic selective pressure, whereby antigen-positive tumor cells are preferentially eliminated while antigen-negative or antigen-low subpopulations survive and expand. In GB, this mechanism has been particularly well documented for EGFRvIII. Following EGFRvIII-directed CAR-T-cell therapy, decreased EGFRvIII expression was observed in five of seven patients with available post-infusion tumor tissue [119]. Similarly, preclinical IL-13Rα2-CAR-T studies demonstrated tumor recurrence associated with antigen-negative escape variants [120]. Loss or reduced expression of the target antigen may prevent productive recognition of tumor cells by CAR-T cells, thereby diminishing or abolishing therapeutic cytotoxicity. This limitation is particularly relevant to single-antigen-targeting approaches and provides a rationale for the development of CAR-T-cell strategies capable of recognizing multiple antigens.

Independent of CAR-T-cell treatment, EGFRvIII status may also change during GB progression. In paired primary and recurrent tumors, newly detected EGFRvIII positivity at recurrence has been reported, although this pattern appears uncommon, and loss of EGFRvIII positivity is more frequently observed [121,122]. These observations further emphasize that temporal antigen instability can complicate the selection of a durable single-antigen CAR-T target.

Importantly, impaired CAR-T-cell recognition may also occur despite preserved antigen expression when the target epitope becomes functionally inaccessible, for example, through glycosylation-dependent masking or glycocalyx remodeling, as discussed in Section 4.3.

### 4.3. Glycocalyx Remodeling and Glycosylation-Dependent Epitope Masking

The potential contribution of the tumor-cell glycocalyx to impaired CAR-T-cell recognition in GB should also be considered. The glycocalyx is a cell-surface layer composed of glycoproteins, proteoglycans, and glycolipids, whose composition and physical properties can be remodeled in tumor cells [59,75]. In GB, a bulky glycocalyx–integrin feedback loop has been shown to promote a mesenchymal-like phenotype, supporting the biological relevance of glycocalyx remodeling in this disease [75]. Whereas antigen heterogeneity and antigen loss reduce target availability through changes in antigen expression, the glycocalyx may impair CAR-T-cell recognition despite preserved antigen expression.

Changes in the extracellular and glycosylated surface environment of GB have also been documented in patient-derived material. Comparative matrisome and glycoproteomic analyses of human GB tissue identified alterations in matrisomal molecules and glycopeptide features relative to control brain tissue [123], while proteomic profiling further demonstrated extracellular matrix remodeling together with altered glycoprotein signatures [124]. Although these findings do not directly demonstrate glycocalyx-mediated restriction of CAR-T-cell recognition in GB, they support extensive remodeling of the extracellular and glycoprotein environment of GB cells.

Another mechanism that may limit the effectiveness of CAR-T therapy in GB is glycosylation-dependent epitope masking. The barrier formed by the glycocalyx should be considered at a global level, whereas glycosylation-dependent epitope masking acts at the level of individual surface antigens. Glycans located within or close to an epitope may restrict antibody-derived recognition through steric effects or glycan-associated conformational changes [125]. Since CARs use antibody-derived antigen-recognition domains, such masking may leave the antigen present at the cell surface while reducing its functional accessibility to CAR-T cells.

In the context of GB, glycosylation-dependent epitope masking is particularly relevant because several commonly investigated CAR-T target antigens are glycosylated surface molecules. N-glycosylation influences the conformation and membrane orientation of the EGFR ectodomain [126], is required for optimal functional activity of IL-13Rα2 [127], and regulates the surface localization and immunological function of B7-H3 [128]. These observations do not yet demonstrate glycosylation-dependent resistance to CAR-T-cell therapy in GB, but they identify plausible mechanisms by which target presentation or epitope accessibility may be altered. Accordingly, glycocalyx remodeling and glycosylation-dependent epitope masking may jointly reduce functional recognition of surface antigens by CAR-T cells. Studies in other tumor models indicate that glycan-directed interventions can enhance cytotoxic immune-cell engagement or CAR-T-cell activity [59,105]. These findings provide a rationale for investigating such strategies in GB.

### 4.4. Immunosuppressive Tumor Microenvironment

In analyzing the factors limiting the effectiveness of CAR-T therapy in patients with GB, it is also necessary to mention the strongly immunosuppressive TME, which is characterized by the presence of multiple mechanisms inhibiting T cell function.

One such mechanism in the TME of GB patients is the high activity of myeloid cells with an immunosuppressive phenotype, which can secrete factors that directly inhibit T cell proliferation and cytotoxic activity and lead to T cell exhaustion. Such cells may include, for example, microglia or macrophages, which constitute the majority of inflammatory infiltrating cells and may secrete, among others, TGF-β and IL-10 [129].

Moreover, in some tumor types, including glioma, a high expression level of immune checkpoint ligands has also been confirmed. One such ligand is PD-L1, which, through interaction with PD-1 receptors located on the surface of T cells, can functionally inactivate CAR-T cells by blocking T cell activation within the tumor microenvironment [130].

Another mechanism limiting T cell activity is the creation of a microenvironment unfavorable for T cell function due to the specific tumor metabolism. As mentioned earlier, chronic or cyclic hypoxia in GB promotes intense glycolysis in tumor cells, resulting in lactate accumulation, microenvironmental acidification, and nutrient depletion, which collectively impair the cytotoxic activity of effector cells. Moreover, ATP released by GB cells into the extracellular space is enzymatically converted into adenosine, which, via the A2AR receptor on T cells, can suppress the immune response induced by them [131].

The described mechanisms of immunosuppressive activity of the TME result in strong inhibition of CAR-T effector function in GB and may constitute one of the reasons for CAR-T therapy failure not only in patients with GB but also in patients diagnosed with other types of central nervous system (CNS) tumors.

### 4.5. Blood–Brain Barrier and Restricted CAR-T-Cell Trafficking

Another mechanism by which CAR-T activity is significantly limited in GB patients is the blood–brain barrier (BBB). The BBB is a structure composed of endothelial cells, pericytes, and astrocytic end-feet, which regulates the transport of both cells and molecules between the circulatory system and the CNS [132]. The BBB is highly selective and restricts the migration of immune cells into the brain.

Under physiological conditions, T cell transport across the BBB is a tightly regulated process that requires activation of adhesion pathways and chemokine signaling [133,134]. In patients with GB, partial disruption of BBB integrity has been observed; however, this barrier remains functionally heterogeneous and may still limit CAR-T access to certain tumor regions, resulting in the formation of tumor niches in which cells are inaccessible to immunotherapy [132].

Moreover, limited migration and accumulation of CAR-T cells within the tumor microenvironment may also result from reduced expression of adhesion molecules and chemotactic factors that would otherwise facilitate T cell migration through the brain endothelium. Such limited migration across the BBB should be particularly considered in situations where CAR-T cells are administered intravenously, since, in intratumoral or intraventricular administration, there is no need to cross the BBB, as the cells are delivered directly into the CNS.

## 5. Glycosylation and Other Post-Translational Modifications of Key CAR-T Targets as Barriers to CAR-T Efficacy

Post-translational modifications (PTMs) of tumor-associated antigens represent an important but still underappreciated mechanism that may limit the efficacy of CAR-T cell therapy [104]. In contrast to TCRs, which recognize processed peptide–MHC complexes, CARs bind intact surface antigens through antibody-derived scFv domains. Consequently, antigen conformation, glycosylation, proteolytic shedding, internalization, and other PTM-associated changes may influence CAR binding, functional target accessibility, and the stability of immune synapse formation. Such alterations may mask epitopes, modify protein conformation, reduce surface target density, or impair productive scFv–antigen interactions even when the antigen remains detectable on the cell surface [104,125,135].

The most extensively characterized example of glycosylation-dependent impairment of CAR recognition involves the CD19 antigen in B-cell malignancies [104,136]. Loss of the Golgi-resident protease SPPL3 leads to hyperglycosylation of CD19, including additional N-glycans at asparagine N125, which directly impairs binding of the commonly used FMC63-derived CAR construct [104]. Altered glycosylation or trafficking has also been implicated in the regulation of other B-cell targets, including CD22 [137]. In solid tumors, including GB, aberrant glycosylation and profound tumor heterogeneity may further complicate the functional accessibility of surface antigens, particularly for highly glycosylated therapeutic targets such as HER2, MSLN, MUC1, and EGFR-family receptors [138]. However, the clinical frequency of isolated PTM-mediated escape remains difficult to quantify because antigen-low or antigen-negative relapse may also involve genetic alterations, alternative splicing, transcriptional regulation, or therapeutic selection of antigen-negative tumor populations [139,140,141,142,143].

Building on these observations, similar principles may be relevant to CAR-T-cell therapy in GB. Beyond genetic loss or heterogeneous expression of target antigens, the biochemical organization of the tumor cell surface—particularly glycosylation of membrane proteins, glycocalyx architecture, receptor trafficking, and antigen shedding—may influence CAR scFv recognition and the persistence of productive effector–target interactions. The following subsections, therefore, examine selected CAR-T targets relevant to GB, distinguishing experimentally demonstrated molecular effects from mechanisms that remain biologically plausible but have not yet been directly validated as causes of CAR-T-cell resistance in this disease (Table 2).

**Figure 3:**
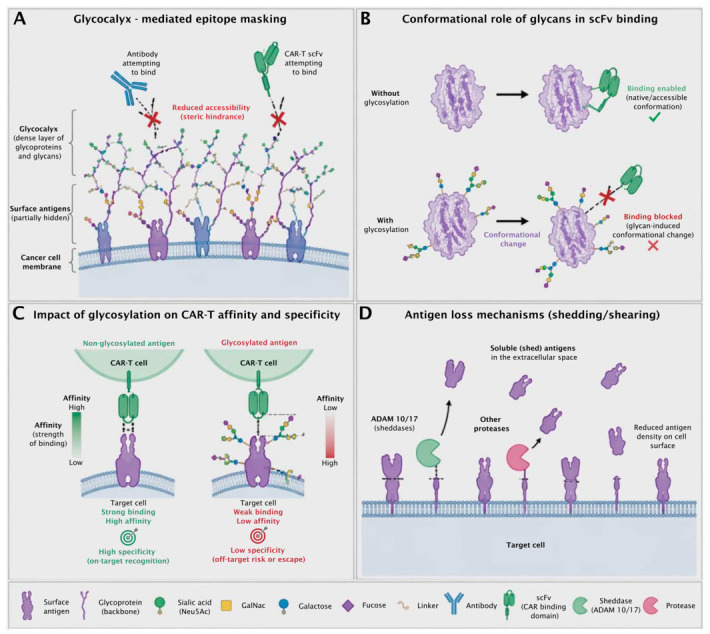
Glycosylation- and shedding-mediated mechanisms that may limit CAR-T-cell efficacy in solid tumors. In Panel (**A**), glycocalyx-mediated epitope masking reduces CAR-T cell access to target antigens on tumor cells. Panel (**B**) illustrates that glycan-dependent conformational effects modulate scFv binding by either facilitating or hindering antigen recognition. In Panel (**C**), altered glycosylation influences CAR-T cell affinity and specificity toward tumor-associated antigens. Panel (**D**) demonstrates that antigen shedding may lead to loss of surface targets and generation of soluble decoys that impair CAR-T cell activity. Arrows indicate the direction of the depicted glycosylation- or shedding-related processes, whereas colors and symbols are defined in the figure legend.

Together, these target-specific examples illustrate how glycosylation, altered surface organization, receptor trafficking, and antigen shedding may reduce functional antigen accessibility and thereby limit productive CAR-T-cell engagement in solid tumors, including GB (Figure 3).

### 5.1. EGFRvIII/EGFR

EGFR and EGFRvIII are membrane proteins whose extracellular-domain glycosylation may influence receptor folding, stability, and epitope exposure, thereby affecting the accessibility of epitopes recognized by antibodies and CAR scFv domains. N-glycosylation may regulate the three-dimensional structure of EGFR and consequently modulate its interactions with ligands [126].

Glycosylation of EGFR may directly affect interactions with the scFv domain by altering the conformation of the receptor extracellular domain. Both the presence and distribution of N-glycans may influence receptor conformation, activation, and dimerization [126,144]. Such conformational changes may result in suboptimal scFv binding, reduced binding affinity, and impaired formation of a stable immunological synapse.

The accessibility of EGFR as a molecular target is also influenced by receptor internalization and shedding. Following ligand binding, EGFR is activated and subsequently undergoes endocytosis, followed by transport to endosomes or lysosomes [157]. Unlike EGFR, EGFRvIII lacks ligand-binding capability and therefore remains at the cell membrane for a longer period [158]. Glycosylation may therefore regulate both epitope accessibility and receptor retention at the cell membrane. It may also influence receptor stability and interactions with components of the endocytic machinery and surface lectins, thereby affecting membrane retention and receptor trafficking [159,160,161].

Shedding of the EGFR extracellular domain is enhanced in tumor cells and depends on metalloproteinase activity. This process may reduce antigen availability at the cell surface and generate soluble forms that could potentially compete for CAR binding [135,162].

Recently, Li et al. described a multimodal immunotherapeutic strategy for glioblastoma that combines oncolytic virus-mediated delivery of tumor antigens CD19 and EGFRvIII with bispecific CAR-T and CAR-NK cells. This platform was designed to address major limitations of CAR-based therapy in GB, including antigen heterogeneity and low surface target expression, by enhancing tumor targeting and sustaining antitumor immune activity [163].

### 5.2. IL-13Rα2

IL-13Rα2 is a membrane protein whose extracellular domain undergoes N-glycosylation, which may influence receptor stability and the spatial organization of extracellular binding regions [127]. To date, glycan-mediated epitope masking of IL-13Rα2 has not been extensively characterized in the context of CAR-T-cell therapy. Nevertheless, glycosylation of cytokine receptors can influence the accessibility and functional organization of extracellular binding domains through changes in protein structure [127,164].

N-glycosylation is functionally relevant to the extracellular organization and ligand-binding properties of IL-13Rα2. Kioi et al. demonstrated that glycosylation of the IL-13Rα2 extracellular domain is required for optimal IL-13 inhibitory activity, whereas He et al. subsequently showed that IL-13 binding depends on each of the four N-glycosylation sites within the IL-13Rα2 extracellular domain and is reduced following mutation or enzymatic/pharmacological disruption of these sites [127,165]. These findings indicate that the glycosylation state of IL-13Rα2 can modify its extracellular functional architecture. Although direct evidence that N-glycosylation alters scFv binding or IL-13Rα2-directed CAR-T-cell efficacy in GB is currently lacking, glycosylation-dependent changes in receptor conformation provide a plausible mechanism by which target accessibility could be modulated despite preserved surface expression.

In addition to its potential effects on receptor conformation, the surface availability of IL-13Rα2 may be influenced by proteolytic release. Chen et al. demonstrated that matrix metalloproteinase-8 (MMP-8) can promote the solubilization of membrane-associated IL-13Rα2, resulting in the generation of soluble receptor forms [166]. Although this finding was obtained outside GB models and has not been evaluated in the context of CAR-T-cell therapy, it provides evidence that IL-13Rα2 surface availability can be regulated by proteolytic processing. As discussed above for EGFR, soluble forms of a target antigen may theoretically reduce productive CAR engagement by decreasing membrane-retained target abundance and/or acting as soluble decoys. Accordingly, soluble IL-13Rα2 forms may represent an additional barrier to IL-13Rα2-directed CAR-T-cell activity in GB; however, this possibility requires direct experimental validation.

### 5.3. Mesothelin

As discussed above for antigen shedding more generally, mesothelin (MSLN), a common CAR-T-cell target in solid tumors and an emerging immunotherapeutic target in human glioblastoma, provides a target-specific example in which proteolytic shedding may reduce the availability of membrane-retained epitopes for immune targeting [149,167,168,169]. MSLN has been investigated as a target antigen for CAR-T-cell therapy in mesothelioma, lung cancer, ovarian cancer, and other malignancies because of its high expression on tumor cells and limited physiological expression on mesothelial cells. Nonetheless, fatal on-target/off-tumor toxicity associated with high-affinity MSLN-targeting CAR-T cells has been reported in clinical studies [170]. Despite numerous clinical trials, no FDA-approved antibody-based therapy targeting MSLN is currently available. Because MSLN is shed from the cell surface through proteolytic cleavage close to the membrane, Liu et al. identified the major proteolytic cleavage sites in MSLN and developed the monoclonal antibody 15B6, which binds to the membrane-proximal protease-sensitive region and inhibits MSLN shedding. The scFv derived from mAb 15B6 may therefore represent an attractive component of MSLN-directed CAR constructs [168]. Preclinical studies have demonstrated that CAR-T cells targeting membrane-proximal epitopes absent from shed MSLN exhibit significantly greater antitumor activity than constructs directed against epitopes present in the soluble domain, underscoring the detrimental effect of shedding on CAR-T-cell engagement and function. Importantly, glycosylation may regulate this process: both N- and O-linked glycans can protect the antigen from proteolytic cleavage, whereas specific glycan patterns may facilitate shedding [150]. Thus, understanding and potentially modulating glycosylation may enhance CAR-T-cell efficacy by stabilizing membrane-associated antigen expression and reducing the impact of soluble fragments.

MSLN is extensively post-translationally modified, with three conserved N-linked glycosylation sites occupied by complex N-glycans that are essential for structural integrity and may also modulate epitope accessibility. Structural analyses indicate that the rigid N-terminal domain is targeted by most therapeutic antibodies, including SS1 and MORAb-009, whereas glycosylation may influence binding, particularly at conformation-sensitive epitopes. In addition to N-glycosylation, O-linked glycosylation contributes to the epitope microenvironment, and tumor-associated alterations in glycosylation patterns may affect the affinity of CAR-derived scFv domains [171]. Consistent with this concept, CAR-T cells targeting membrane-proximal regions of MSLN have shown superior antitumor activity compared with those directed against membrane-distal epitopes, highlighting epitope location and glycan shielding as important determinants of therapeutic efficacy. Zhang et al. demonstrated that meso3 CAR-T cells targeting the membrane-proximal region III of MSLN exhibit superior antitumor activity compared with meso1 CAR-T cells directed against the membrane-distal region I in MSLN-positive solid tumors [172]. Human mesothelin contains three predicted N-linked glycosylation sites, and glycan attachments at these sites have been structurally identified and may influence antibody interactions with the protein. CAR-T cells targeting the N-terminal region (residues 296–390) or the glycosylated C-terminal region (residues 487–598) displayed differential tumor-cell killing, suggesting that the glycosylation state of MSLN may influence T-cell recognition [171].

MSLN has also been shown to undergo antigen internalization following antibody engagement, with the surface protein relocating into intracellular vesicles after ligand binding, consistent with its classification as an internalizing antigen. Another phenomenon that may limit the efficacy of MSLN-targeted CAR-T cells is antigen escape. In this context, high-affinity MSLN-directed CAR-T cells may induce rapid internalization of the antigen from the tumor-cell surface. Together with proteolytic shedding, antigen internalization may reduce surface antigen availability and shorten the duration of effective CAR-T signaling. This example illustrates how altered glycosylation states may modulate endocytosis and membrane persistence by dynamically regulating surface antigen levels [169]. Yang et al. demonstrated that high-affinity MSLN CARs were associated with fatal on-target/off-tumor toxicity, whereas affinity-tuned CARs rendered T cells more selective for MSLN-high tumors. The use of lower-affinity CARs may therefore improve discrimination between cells with high and low MSLN expression, thereby limiting unintended recognition of normal cells expressing lower levels of this antigen [170].

### 5.4. B7-H3

B7-H3 (CD276), an immunoregulatory member of the B7 family, has emerged as an attractive target for CAR-T-cell therapy in glioblastoma and other solid tumors because it meets several important criteria of a tumor-associated antigen. Nevertheless, as with most targets investigated in GB, B7-H3-directed therapy does not overcome all major barriers to effective treatment. In a glioblastoma xenograft model, novel B7-H3-targeted CAR-T cells demonstrated potent antitumor activity, supporting further translational development of this therapeutic strategy [173]. B7-H3 is frequently overexpressed in aggressive tumor cells, while its expression is limited or absent in many normal tissues, and it is extensively modified by glycosylation, which may regulate protein stability, localization, and immune interactions [174,175,176].

More specifically, PRIDE-based analysis in colorectal cancer demonstrated differentiation-associated CD276 glycosylation patterns, with denser glycosylation within the IgV and IgC domains of epithelial-like tumors and sparser, more membrane-proximal glycosylation in mesenchymal-like tumors [176]. Although these findings were obtained outside the GB setting, they suggest that the glycosylation state of B7-H3 may influence its structural organization and functional accessibility on tumor cells.

Clinical trials have investigated B7-H3-targeted CAR-T cells in refractory or recurrent GB, diffuse intrinsic pontine glioma (DIPG), neuroblastoma, medulloblastoma, ependymoma, and metastatic brain tumors [177]. However, although B7-H3-targeted nanoCAR-T cells showed promise in preclinical GB models, in vivo toxicity was observed. Off-tumor recognition of low-level B7-H3 expression in healthy murine tissue by the cross-reactive nanoCAR-T cells was identified as a potential cause of this toxicity. These findings highlight the need for caution when designing highly sensitive B7-H3-targeted CAR-T-cell approaches [178].

### 5.5. HER2

Human epidermal growth factor receptor 2 (HER2), another member of the ERBB receptor family alongside EGFR, has been identified as a clinically relevant target for CAR-T cell therapy in glioma. Early-phase clinical evaluation has demonstrated safety and signs of therapeutic activity in patients with HER2-positive CNS tumors, including gliomas [179]. HER2-targeted approaches have also been investigated in preclinical models of glioma and diffuse midline glioma [180,181]. HER2 overexpression has been reported in approximately 80% of GB cases [182]. In the context of HER2-targeted CAR-T-cell therapy, therapeutic efficacy may be limited not only by heterogeneous antigen expression between tumors, but also by post-translational regulation and dynamic presentation of HER2 at the tumor-cell surface. HER2 undergoes N-linked glycosylation, which may modulate epitope accessibility to scFv domains and thereby influence both binding affinity and the stability of scFv–target interactions [183]. In addition, internalization of the HER2–CAR complex may reduce the duration of antigen exposure at the cell surface and, together with glycosylation-dependent epitope masking, limit efficient CAR-T-cell recognition and cytotoxic function.

Evidence from other HER2-positive tumor settings further indicates that glycosylation may modulate antibody-based target recognition. In HER2-positive gastric cancer cells, site-specific N-glycan analysis revealed the involvement of α2,6-sialic acids on HER2 N-glycans in regulating sensitivity to trastuzumab, a well-known anti-HER2 antibody. Duarte et al. demonstrated that the sialyltransferase ST6GAL1 specifically targets N-glycosylation sites within the trastuzumab-binding domain of HER2. Loss of ST6GAL1 expression reshaped both the cellular and HER2-specific glycomes, prolonged HER2 half-life at the cell surface, and sensitized HER2-dependent gastric cancer cells to trastuzumab-induced cytotoxicity through stabilization of ErbB dimers at the plasma membrane [184].

Glioma cells may exhibit relatively low HER2 surface density or release the receptor extracellular domain, thereby reducing target availability at the tumor-cell surface and facilitating escape from CAR-T-cell surveillance. In a phase I clinical study, Ahmed et al. demonstrated the safety of HER2-directed CAR-T-cell therapy in progressive GB, although durable clinical responses remained limited [155]. One potential contributing factor is the relatively low density of HER2 expression on GB cells compared with strongly HER2-overexpressing tumors, such as breast cancer, which may impair efficient CAR-T-cell activation and sustained antitumor activity [154]. To address antigen heterogeneity and escape, Hegde et al. engineered a bispecific tandem CAR (TanCAR) targeting HER2 and IL-13Rα2. TanCAR T cells were able to recognize either antigen and lyse autologous GB cells, thereby reducing antigen escape and enhancing antitumor efficacy. In a mouse model, TanCAR T cells prolonged survival and demonstrated improved therapeutic activity through simultaneous targeting of both antigens [185].

### 5.6. GD2

Disialoganglioside GD2 is a glycosphingolipid highly enriched in the plasma membrane. It is strongly associated with neural crest-derived tumors and has also been detected in subsets of glioblastoma. Unlike protein-based antigens, GD2 is embedded within the lipid-rich glycocalyx, and its accessibility is strongly influenced by membrane organization, lipid raft composition, and local glycosylation architecture [186]. Thus, GD2 represents a glycolipid-defined CAR-T-cell target for which antigen recognition depends less on classical protein epitope masking and more on glycocalyx density, membrane topology, and ganglioside clustering.

Immunotherapeutic strategies targeting GD2, including anti-GD2 antibodies and anti-GD2 CAR-T cells, are currently being investigated in combination with cytokines, chemotherapy, and immune checkpoint blockade [155,187,188,189,190]. Importantly, unlike mesothelin, GD2 is not a protein and therefore does not undergo classical proteolytic shedding. Nevertheless, it may be redistributed or internalized within membrane microdomains, thereby contributing to functional antigen modulation [191,192,193]. Moreover, Yesmin et al. demonstrated that GD2 can be released in extracellular vesicles (EVs) [156].

An additional clinically relevant limitation is the expression of GD2 in normal nervous system tissues, which is associated with a risk of on-target/off-tumor toxicity [194]. In a meta-analysis of eight studies involving 146 patients with neuroblastoma, Habibi et al. reported the pooled complete response (CR) and partial response (PR) rates of 39.57% and 15.83%, respectively. These findings suggest moderate clinical activity of GD2-targeted CAR-T cells, although hematologic toxicities were reported [194]. In addition, substantial heterogeneity in treatment response has been observed among patients with H3K27M-mutated diffuse midline gliomas treated with GD2-directed CAR-T cells [155].

## 6. Emerging Strategies to Overcome Glycosylation- and Shedding-Mediated CAR-T Resistance

As discussed above, glycosylation and antigen shedding represent critical, yet often underappreciated, barriers limiting CAR-T cell efficacy in solid tumors, including glioblastoma. Importantly, these mechanisms may limit CAR-T-cell efficacy either by reducing functional epitope accessibility, as in glycan-dependent masking and glycocalyx remodeling, or by decreasing surface-retained antigen abundance, as in proteolytic shedding. Dense tumor glycocalyx structures, glycan-dependent epitope masking, and proteolytic release of target antigens impair synapse stability and CAR-sustained signaling. These mechanisms contribute to antigen loss, functional exhaustion, and heterogeneous therapeutic responses.

**Figure 4:**
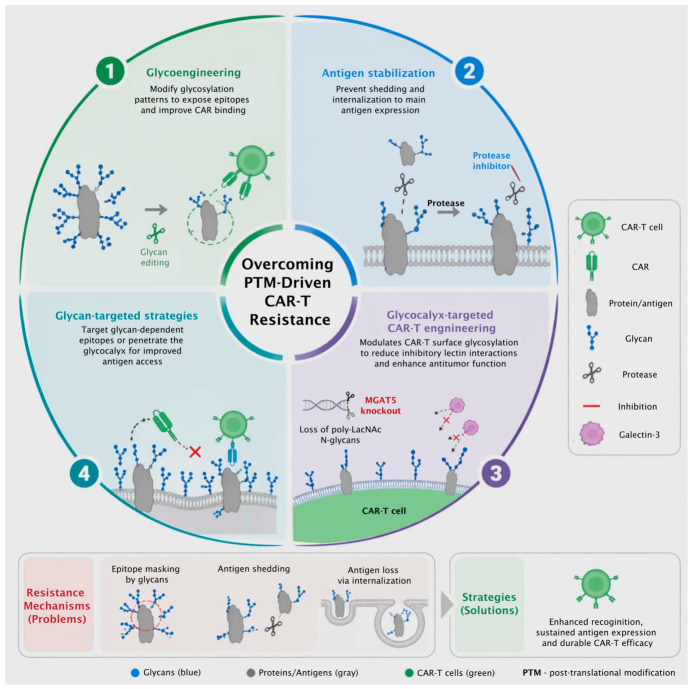
Target-, effector-, and microenvironment-focused strategies to overcome glycosylation- and shedding-mediated resistance and improve CAR-T cell efficacy in solid tumors. Panels 1 and 2 illustrate target-centric interventions that restore epitope accessibility through deglycosylation, sheddase inhibition, or glycocalyx-bridging approaches. In Panel 3, effector-centric glycoengineering enhances CAR-T persistence and resistance to lectin-mediated suppression. Panel 4 demonstrates microenvironment-centric strategies that disrupt glycoimmune checkpoints, improving CAR-T infiltration and cytotoxicity.

To address these challenges, at least three emerging strategies aim to reprogram glycosylation-dependent interactions at multiple levels of the CAR-T axis. First, target-centric approaches focus on modulating glycosylation and proteolytic shedding of tumor antigens to improve epitope accessibility and surface persistence. Second, effector-centric strategies seek to remodel the glycocalyx and glyco-signaling machinery of CAR-T cells themselves, thereby enhancing immune synapse formation and antitumor persistence. Finally, microenvironment-centric interventions target aberrant glycosylation within the tumor microenvironment, including sialylation-driven immune checkpoints and galectin-rich niches that suppress CAR-T function (Figure 4).

### 6.1. Target-Centric Strategies: Modulating Glycosylation and Shedding of CAR-T Antigens

Solid tumors exhibit profound alterations in protein glycosylation, including increased N-glycan branching and heterogeneous glycoform patterns. Early on, Greco and colleagues hypothesized that peptidic epitopes may be sterically masked by glycans, particularly on heavily glycosylated tumor antigens targeted by CAR-T cells. They demonstrated that N-glycans, especially those regulated by the branching enzyme MGAT5, protect tumor cells from CAR-T-mediated killing by interfering with immune synapse formation and reducing T-cell activation. Using CRISPR–Cas9-mediated knockout of *Mgat5*, the authors generated N-glycosylation-defective pancreatic tumor cell lines expressing the heavily glycosylated antigens CD44v6 and CEA. Importantly, combining CAR-T cells with 2-deoxy-D-glucose (2DG), which disrupts tumor-cell N-glycan expression, enhanced antitumor efficacy in preclinical models, supporting a potential combinatorial strategy to overcome glycan-mediated resistance [105,195].

Antigen shedding represents an additional target-centric mechanism limiting CAR-T efficacy, particularly in the context of MSLN. Despite extensive clinical development, none of the MSLN-targeting agents have yet achieved FDA approval, in part due to rapid proteolytic cleavage of the antigen from the tumor cell surface [196]. ADAM10 and ADAM17 have been identified as key “sheddases” responsible for MSLN cleavage [197]. Genetic or pharmacological inhibition of these proteases reduces mesothelin shedding, increases surface antigen density, and enhances the activity of antibody-based therapies. Building on this concept, Chu and colleagues proposed pharmacological inhibition of mesothelin shedding as a strategy to enhance CAR-T cell efficacy. Using ADAM17 inhibitors such as TMI-1, they demonstrated increased surface MSLN expression and improved CAR-T-mediated cytotoxicity in preclinical models, supporting the rationale for combining CAR-T therapy with metalloprotease inhibitors [169]. In parallel, Liu et al. demonstrated an alternative strategy based on epitope selection: CAR-T cells targeting a juxtamembrane region of MSLN were not blocked by shed mesothelin and showed potent antitumor activity in preclinical models, indicating that membrane-proximal epitope targeting may help overcome the functional consequences of antigen shedding [168].

#### Novel Glycocalyx-Bridging Strategies to Overcome Epitope Inaccessibility

In the context of mesothelin-targeted CAR-T therapy, Park and colleagues proposed an alternative strategy that does not directly remove glycans but instead exploits tumor-associated glycosylation to enhance CAR engagement. Recognizing that the expanded tumor glycocalyx, rich in glycoproteins such as MUC1, can sterically hinder CAR access to target antigens, the authors engineered a non-signaling glyco-bridge binder recognizing Tn-MUC1 and incorporated it into mesothelin-directed CAR-T cells. This glyco-bridge increased functional avidity and facilitated CAR activation in a density- and affinity-dependent manner, thereby enhancing tumor recognition and cytotoxicity despite the presence of a dense glycocalyx. This approach reframes aberrant tumor glycosylation from a barrier into a functional scaffold for CAR-T engagement [198].

### 6.2. Effector-Centric Strategies: Engineering the CAR-T Cell Glycocalyx

Beyond tumor-intrinsic glycosylation, the glycocalyx of CAR-T cells themselves has emerged as a modifiable determinant of function. De Bousser et al. demonstrated that deletion of the *MGAT5* gene in human CAR-T cells abolishes poly-LacNAc N-glycan structures on the CAR-T surface. This glycoengineering approach resulted in improved tumor control in carcinoma and lymphoma models, enhanced CAR-T cell persistence, and increased antitumor activity. Notably, this technology has progressed toward intellectual property protection, underscoring its translational potential [199,200].

In parallel, strategies aimed at reducing CAR-T susceptibility to inhibitory lectin signaling have gained attention. Lau and colleagues identified galectin-3 (Gal-3) as a key extrinsic suppressor of CAR-T cell function and demonstrated that modulation of α2,6-linked sialylation on CAR-T cell surfaces enhances resistance to galectin-rich, immunosuppressive microenvironments. Together, these findings support the concept that remodeling the CAR-T glycocalyx can directly enhance immune synapse formation, signaling robustness, and persistence [201].

### 6.3. Microenvironment-Centric Strategies: Targeting Aberrant Glycosylation in the TME

The TME is characterized by widespread glycosylation abnormalities that actively suppress immune responses. Tumor-associated hypersialylation generates a dense, highly negatively charged glycocalyx that sterically masks tumor-associated antigens, destabilizes immune synapses, and engages inhibitory Siglec receptors on immune cells, collectively impairing CAR-T activation and cytotoxicity.

Several approaches aim to therapeutically exploit this vulnerability. Sirini et al. investigated N-glycosylation blockade in TME cells in models of colorectal cancer and pancreatic ductal adenocarcinoma liver metastases treated with CEA-specific CAR-T cells. Their work demonstrated that inhibiting N-glycosylation can alleviate multiple barriers to CAR-T efficacy by acting not only on tumor cells, but also on immunosuppressive stromal and myeloid populations [202].

In a complementary strategy, Gray and colleagues developed an antibody–sialidase conjugate targeting HER2 that selectively removes sialoglycans from tumor cell surfaces. This localized desialylation enhanced immune cell infiltration, activation, and survival in vivo, in a manner dependent on Siglec-E signaling in tumor-infiltrating myeloid cells. These findings position aberrant sialylation as a druggable glycoimmune checkpoint within the TME [203].

Together, these complementary approaches highlight glycosylation as a therapeutically actionable layer of regulation (Figure 4). By shifting the focus from antigen expression to functional epitope availability, glycoengineering strategies offer a rational framework to overcome resistance mechanisms in the context of immunotherapies in solid tumors.

## 7. Redefining CAR-T Target Accessibility—Future Perspectives on Overcoming CAR-T Limitations

Unlike TCR-mediated recognition, which is directed toward processed peptide–MHC complexes and includes only a limited fraction of post-translationally modified presented peptides, CARs bind intact surface antigens whose extracellular epitopes may be directly affected by glycosylation and other PTMs [204]. Although PTMs rarely act in isolation and frequently coexist with genetic and splicing alterations, they may contribute to therapeutic resistance and relapse after CAR-T-cell therapy [140,141,142,143], particularly in cases where target-antigen expression is retained but functional recognition is impaired. This section summarizes current approaches to detect, interpret, and therapeutically address PTM-mediated immune escape, highlighting emerging strategies for integrating PTM monitoring into precision CAR-T-cell therapy.

In general, two complementary approaches are available: detection of PTM-associated changes in patient material and subsequent adaptation of CAR designs to overcome these modifications, as described above.

Detecting PTMs in patient material is feasible, though not yet routine. Pre- and post-relapse samples (bone marrow, biopsies, peripheral blood, or CSF) can be analyzed using Western blot or SDS-PAGE for molecular weight shifts, mass spectrometry-based glycomics or glycoproteomics for detailed glycan profiling, lectin blotting, or flow cytometry with glycan-specific reagents. Moreover, single-cell multi-omics approaches can be used to identify subclones with altered PTM patterns [205,206]. SPPL3 status should also be considered [207]. These correlative studies in academic trials help distinguish PTMs from other escape routes and guide subsequent therapy choices. Advances in understanding these modifications are accelerating through improved omics technologies and reverse translational research from clinical relapses. Patient-derived material, such as bone marrow aspirates, lymph node biopsies, peripheral blood, or cerebrospinal fluid collected before lymphodepletion and at relapse, can be interrogated with advanced techniques.

To proactively eliminate or minimize PTM-related escape, CAR-T engineering has progressed slowly. One approach involves developing novel scFvs, nanobodies, or DARPins with reduced sensitivity to glycosylation or other modifications, often through yeast display libraries or structure-guided design that accommodates hyper- or hypoglycosylated forms [208]. Glycan-specific CARs (e.g., targeting tumor-associated Tn-MUC1 or sialyl-Tn) turn cancer-specific PTMs into an advantage rather than a liability [208]. Pharmacologic agents can meaningfully reduce key PTMs that drive CAR-T resistance [105,209,210,211].

In hematological malignancies, the combination of γ-secretase inhibitors with BCMA CAR-T is the most clinically validated example, with encouraging response rates and mechanistic proof that restoring antigen density overcomes shedding [212]. For glycosylation issues (e.g., CD19), inhibitors like kifunensine provide a strong preclinical rationale and are likely to advance into translational studies [104,213]. As data from ongoing experiments (2025–2026) emerge, these strategies may become a standard adjunct to broaden treatment options not only in hematologic malignancies but also in solid tumors. Unfortunately, many glycosylation or secretase inhibitors affect normal cells (e.g., causing gastrointestinal or hematologic side effects), so short-term “run-in” dosing before or during CAR-T infusion is preferred over continuous use [214]. However, instead of directly counteracting post-translational modifications, dual- or trispecific CAR-T cells (tandem, co-expressed, or logic-gated AND/OR systems) can be designed to simultaneously target multiple antigens (e.g., mesothelin/B7-H3) [215,216,217].

Selecting antigens such as gangliosides can provide another option. In this scenario, escape via PTMs on one antigen does not confer full resistance. Still, emerging concepts also explore “glycan shielding” (e.g., *SPPL3* knockout in allogeneic CAR-T cells to modulate immunogenicity) and integration with epigenetic or metabolic interventions that influence PTM landscapes [104].

As single-cell and glycoproteomic technologies mature, PTM monitoring may become a standard component of precision CAR-T management. In the context of CAR-T therapy, such profiling would provide insight into how biochemical remodeling of tumor antigens and the surrounding glycocalyx affects CAR scFv binding, immune synapse formation, and effector persistence. Ultimately, PTM-aware analyses could support rational target selection, guide the engineering of CAR constructs resilient to antigenic remodeling, and enable adaptive therapeutic strategies tailored to the evolving tumor phenotype.

In parallel, disulfidptosis should be considered an emerging, context-dependent metabolic–redox vulnerability in GB. Although this form of cell death can be experimentally induced in GB under defined metabolic conditions, whether disulfidptosis-oriented interventions can enhance CAR-T-cell-mediated tumor control remains unknown and requires direct experimental evaluation.

## Figures and Tables

**Table 1:** Comparison of regulated cell death modalities relevant to glioblastoma and their potential relationship to CAR-T-cell therapy.

Regulated Cell Death Modality	Defining Trigger/Execution Mechanism	Characteristic Molecular or Structural Lesion	Key Distinction from Disulfidptosis	Evidence/Relevance in GB	Potential Relationship to CAR-T-Cell Therapy	Key References
Disulfidptosis	Excessive intracellular disulfide stress under limited NADPH-dependent reducing capacity, particularly in SLC7A11-high cells exposed to glucose starvation	Aberrant disulfide bonding in actin cytoskeletal proteins and collapse of the F-actin network	Defining feature: redox-dependent collapse of the actin cytoskeleton	Experimentally demonstrated in GB cells following TrxR1 inhibition under glucose-starved conditions; TrxR1 depletion reduced tumor growth in an orthotopic xenograft model	No direct evidence currently demonstrates that disulfidptosis-oriented interventions enhance CAR-T-cell-mediated tumor control in GB	[21,22]
Apoptosis	Intrinsic mitochondrial or extrinsic death-receptor signaling leading to caspase activation	Caspase-mediated cellular dismantling, with preserved membrane integrity during early execution	Caspase-dependent execution rather than disulfide-stress-induced actin collapse	Resistance to apoptotic signaling contributes to treatment resistance in GB	CAR-T cells can induce apoptotic killing through perforin/granzyme B and death-receptor pathways, including Fas/FasL	[24,26]
Ferroptosis	Failure of lipid peroxide control involving iron-dependent oxidative damage and dysfunction of system $x_{c}^{-}$/GSH/GPX4-associated antioxidant protection	Accumulation of lethal lipid peroxides	Iron-dependent lipid peroxidation rather than cytoskeletal disulfide damage	Ferroptosis-related metabolic vulnerabilities and immune associations have been reported in glioma and GB	Cytokine-dependent signaling from activated effector T cells, particularly IFN-γ-mediated regulation of system $x_{c}^{-}$, can promote tumor-cell ferroptosis; direct relevance to CAR-T therapy in GB remains unconfirmed	[24,27]
Necroptosis	RIPK1/RIPK3/MLKL-dependent signaling, typically engaged when apoptotic execution is impaired or blocked	MLKL-mediated plasma membrane permeabilization and lytic cell death	Kinase-mediated membrane disruption rather than actin disulfide collapse	Necroptosis-related signaling has been reported in glioma/GB models, but its therapeutic significance remains incompletely defined	Potentially relevant to inflammatory tumor-cell death, but direct involvement in CAR-T responses in GB has not been established	[24]
Pyroptosis	Caspase- or granzyme-dependent cleavage of gasdermins leading to membrane pore formation	Gasdermin-mediated membrane pores and inflammatory cell lysis	Gasdermin-dependent pore formation rather than cytoskeletal disulfide damage	Pyroptosis-related mechanisms have been investigated in GB, but evidence in GB immunotherapy settings remains limited	CAR-T-derived granzyme B can induce caspase-3/GSDME-mediated pyroptosis in target tumor cells; its relevance to GB-directed CAR-T therapy remains unknown	[24,28]

**Table 2:** Glycosylation-driven regulation of CAR-T target accessibility and therapeutic response in glioblastoma.

CAR-T Target	Glycosylation Role in GB	Dominant Effect on Antigen Biology	Evidence Type	Therapeutic Implication for CAR-T	References
EGFR/EGFRvIII	N-glycosylated ectodomain; heterogeneous glycoforms reported	Alters receptor conformation, surface organization, and steric accessibility within the glycocalyx	in vitro binding studies, tumor slice imaging	affinity tuning; targeting exposed ectodomain epitopes; combination with microenvironment modulation	[126,144,145]
IL-13Rα2	N-glycosylated receptor; GB-overexpressed	Glycosylation influences folding and receptor stability; indirect effects on ligand-binding interface	receptor biology, immunotoxin/CAR studies	CAR affinity optimization; avidity engineering; improved synapse formation	[146,147,148]
Mesothelin	N- and O-glycosylated; tumor-associated glycoforms	Dominant regulation via proteolytic shedding resulting in reduced surface antigen density	in vitro, xenograft models	targeting membrane-retained epitopes; resistance to soluble antigen sink	[149,150]
B7-H3 (CD276)	Highly N-glycosylated immune checkpoint-like protein	Glycosylation contributes to structural stability and surface persistence; direct masking not established	tumor expression studies, CAR-T preclinical models	target stable extracellular domains; combination with TME modulation	[151,152]
HER2	Strongly N-glycosylated receptor tyrosine kinase; multiple glycoforms, including sialylated structures reported	Glycosylation modulates receptor conformation, epitope accessibility (trastuzumab-like epitopes), and membrane stability; can influence shedding/internalization dynamics	CAR-T preclinical models, antibody binding studies	epitope selection (membrane-proximal regions), affinity tuning CARs, potential combination with glyco- or sheddase-targeting strategies	[153,154]
GD2	Ganglioside (glycolipid); embedded in membrane glyco-lipid rafts	Antigen presentation depends on membrane organization, lipid raft composition, and glycocalyx architecture rather than epitope exposure; rather no shedding, detection in extracellular vesicles (EVs)	preclinical CAR-T models, neuro-oncology studies	targeting membrane microdomains; risk mitigation for off-tumor neurotoxicity; potential combination with membrane-disrupting strategies	[155,156]

## Data Availability

No new data were created or analyzed in this study.

## Abbreviations

The following abbreviations are used in this manuscript:

6PGD	6-phosphogluconate dehydrogenase
A2AR	adenosine A2A receptor
ADAM	a disintegrin and metalloproteinase
ADAM10	a disintegrin and metalloproteinase domain-containing protein 10
ADAM17	a disintegrin and metalloproteinase domain-containing protein 17
B7-H3	B7 homolog 3
BBB	blood–brain barrier
BCMA	B-cell maturation antigen
CAR	chimeric antigen receptor
CAR-NK	chimeric antigen receptor natural killer cells
CAR-T	chimeric antigen receptor T cells
CD	cluster of differentiation
CD4	cluster of differentiation 4
CD8	cluster of differentiation 8
CD19	cluster of differentiation 19
CD20	cluster of differentiation 20
CD22	cluster of differentiation 22
CD276	cluster of differentiation 276
CEA	carcinoembryonic antigen
CNS	central nervous system
CR	complete response
CRISPR–Cas9	clustered regularly interspaced short palindromic repeats–CRISPR-associated protein 9
DHODH	dihydroorotate dehydrogenase
DIPG	diffuse intrinsic pontine glioma
DNA	deoxyribonucleic acid
DR4	death receptor 4
DR5	death receptor 5
ECM	extracellular matrix
EGFR	epidermal growth factor receptor
EGFRvIII	epidermal growth factor receptor variant III
EGFRwt	wild-type epidermal growth factor receptor
ER	endoplasmic reticulum
EVs	extracellular vesicles
F-actin	filamentous actin
FAK	focal adhesion kinase
FDA	Food and Drug Administration
GB	glioblastoma
G6PD	glucose-6-phosphate dehydrogenase
GD2	disialoganglioside GD2
GLUT	glucose transporter
GLUT1	glucose transporter 1
GPX4	glutathione peroxidase 4
GPRC5D	G protein-coupled receptor class C group 5 member D
GSH/GSSG	reduced glutathione/oxidized glutathione
HBP	hexosamine biosynthetic pathway
HER2	human epidermal growth factor receptor 2
HIF-1α	hypoxia-inducible factor 1 alpha
HK2	hexokinase 2
IDH	isocitrate dehydrogenase
IL-10	interleukin-10
IL-13	interleukin-13
IL-13Rα2	interleukin-13 receptor alpha 2
MDSCs	myeloid-derived suppressor cells
MGAT1	alpha-1,3-mannosyl-glycoprotein 2-beta-N-acetylglucosaminyltransferase
MGAT5	alpha-1,6-mannosyl-glycoprotein 6-beta-N-acetylglucosaminyltransferase
MGMT	O6-methylguanine-DNA methyltransferase
MHC	major histocompatibility complex
MSLN	mesothelin
mTOR	mechanistic target of rapamycin
MUC1	mucin 1
NADP+	nicotinamide adenine dinucleotide phosphate
NADPH	reduced nicotinamide adenine dinucleotide phosphate
NF-κB	nuclear factor kappa B
NK	natural killer
NOX4	NADPH oxidase 4
O-GlcNAc	O-linked N-acetylglucosamine
OGT	O-GlcNAc transferase
PD-1	programmed cell death protein 1
PD-L1	programmed death-ligand 1
PI3K/Akt	phosphoinositide 3-kinase/protein kinase B
PPP	pentose phosphate pathway
PR	partial response
PTM	post-translational modification
Rac	Ras-related C3 botulinum toxin substrate
ROS	reactive oxygen species
scFv	single-chain variable fragment
Siglec	sialic acid-binding immunoglobulin-type lectin
SLC7A11	solute carrier family 7 member 11
SPPL3	signal peptide peptidase-like 3
ST6GAL1	beta-galactoside alpha-2,6-sialyltransferase 1
TAA	tumor-associated antigen
TanCAR	tandem chimeric antigen receptor
TCR	T-cell receptor
TERT	telomerase reverse transcriptase
Tex/T_ex	T-cell exhaustion/exhausted T-cell state
TGF-β	transforming growth factor beta
TME	tumor microenvironment
TNF	tumor necrosis factor
TRAIL	TNF-related apoptosis-inducing ligand
TrxR1	thioredoxin reductase 1
UDP-GlcNAc	uridine diphosphate N-acetylglucosamine
WRC	WAVE regulatory complex
xCT	cystine/glutamate antiporter light-chain subunit
